# Harnessing student involvement: Perspectives from building infrastructure for sexual harassment prevention and response at the University of Lagos, Nigeria

**DOI:** 10.7189/jogh.12.03075

**Published:** 2022-12-16

**Authors:** Abiola Akiyode-Afolabi, Abigail Ogwezzy-Ndisika, Elizabeth Christian, Adaobi Okonkwo, Kate Klein, Sarah Brown, Ngoki Kamau, Robert Murphy, Lifang Hou, Folasade Ogunsola

**Affiliations:** 1 University of Lagos, Lagos, Nigeria; 2 Northwestern University, Evanston, Illinois, USA

Sexual harassment and misconduct, particularly against female students and employees, is a significant problem in tertiary academic institutions in most countries. For students and trainees who rely on mentorship to move their careers forward, reporting harassment and misconduct by a mentor can alter the opportunities made available to them and ultimately their career path. For this reason, there may be underreporting of incidents, as victims fear adverse outcomes to coming forward [1].

In Nigeria, sexual harassment and misconduct is a significant problem; nearly a quarter of women suffers from some form of sexual violence in their lifetime [2]. In academia, this number may be much higher. One study reported that 70% of Nigerian women reported being victims of sexual harassment and misconduct while in tertiary education institutions [3].

In December 2020, the NIH Fogarty International Center (FIC) with support from the Office of Research, awarded supplemental funds to 10 academic institutions in low-middle income countries [4]. This supplement was in response to FIC recognizing that sexual harassment had become an issue that has forced women working in global health to either become stagnant in their career, or change careers entirely [5]. In collaboration with the Robert J. Havey Institute for Global Health, The Women's Center, and The Office for Equity at Northwestern University (NU), the University of Lagos in Nigeria (UNILAG) applied for funds to strengthen institutional resources around prevention of harassment and misconduct already initiated. The timing of this opportunity was ideal, because of the 2019 televised exposé from the British Broadcast Company (BBC) African Eye series titled “Sex for Grades” with UNILAG as one of the focal institutions [6,7]. The BBC exposé reignited efforts to strengthen the policy and improve education and outreach on sexual harassment and misconduct at UNILAG; and create an office to serve as the central resource for victims and survivors. NU and UNILAG have a long history of partnership developing and implementing FIC training grants – since 2009. They have partnered on four in addition to a National Cancer Institute cooperative research project which included a research training component.

This paper focuses on the accomplishments of one of the aims of the supplement; to increase awareness of sexual harassment on UNILAG’s campus. To achieve this aim, the UNILAG team worked with students to develop messaging that could be used campus wide.

**Figure:**
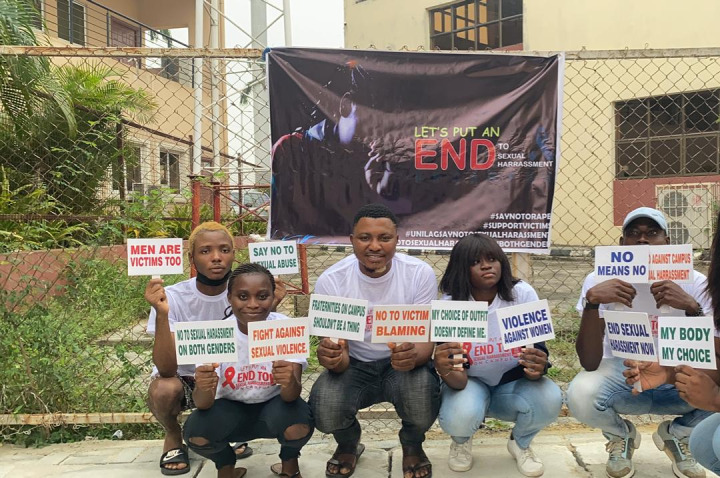
Photo: Students rehearsing their campaign messages (from Abigail Ogwezzy’s collection, used with permission).

Participants were final-year students in a Behavioral Change Communication course, within the UNILAG Mass Communication program. Students had previous training on sensitive issues, such as developing communication for the prevention of the spread of HIV through community outreach work, which places them in a good stead to design sexual harassment awareness slogans for use on campus.

The authors believe that involving students in the creation of the slogans helped democratize the collective effort to prevent sexual harassment at UNILAG by promoting a student-led process in line with a participatory approach to development. The participatory approach stresses that involvement is not merely inclusive of, but largely emanating from, the traditional “receivers” of messages. The participatory approach gave UNILAG students the right as stakeholders to speak against sexual harassment. Furthermore, it facilitated a more equitable exchange of ideas, knowledge, and experiences in the quest to address sexual harassment in tertiary institutions. By creating these slogans, the students became more knowledgeable on how to prevent sexual harassment on campus and became advocates for the cause.

With the goal of raising awareness of sexual harassment prevention, participants prepared slogans and communication materials. Among the messages chosen were: “Don’t be silent, report sexual harassment cases. If you have been sexually harassed, visit UNILAG Counselling Unit”, “Don’t be ashamed of your story …it will help inspire others”, “Speak up! It is not in vain…we must send a message across the world that there is no disgrace in being a survivor of sexual harassment”, “The fight is ours! The struggle is ours!”, “Keep it professional…Stop sexual harassment”, “Don’t cross the line…respect boundaries, stop sexual harassment”, “Dear Lecturer, don’t throw away years of hard work – because you could not keep your hands to yourself…#Be responsible”, “#Stop sexual harassment”.

The student experience to creating the slogans was overall positive. We found that involving students in the process of creating the communications materials reduced the distance between communicators and receivers. This expanded the students’ ability to feel like stakeholders in the conversation around sexual harassment awareness and prevention. They felt listened to, which built their confidence and trust in the University’s position against sexual harassment and reduced social distance between communicators and receivers, between leaders and followers. The students were elated that they are now recognized as stakeholders and right holders, and their views count in the sexual harassment equation. In the search for solution against sexual harassment on campus, students can individually and collectively have a voice in the campaign; can use messages to express themselves and provide direction for the campaign against sexual harassment on campus; and can openly talk about sexual harassment and can seek redress without being victimized by perpetrators.

All materials were developed through the Behavior Change Communication course at UNILAG. Though the slogans have not yet been approved for use on campus, we anticipate that the new awareness program will encourage student-led interventions, ownership, and long-term sustainability of UNILAG’s campaign against sexual harassment. It will also promote an inclusive, participatory approach in the campaign against sexual harassment.

As part of the supplement that funded this work, we also held focus groups sessions with relevant personnel, Deans of Faculty and Heads of Department to determine their understanding of the current sexual harassment policies at UNILAG. Focus groups showed that amongst the Deans of Faculty, Heads of Departments and Units, there is a general knowledge of UNILAG’s policy on sexual harassment, although the level of knowledge differs. These positions are tenured, so it is important that engagements and capacity building on the policy is done annually. This is to ensure that any new person appointed into a new position has a knowledge of the policy. In addition, it will deepen the knowledge of those still in office.

We will look at a few data points to measure the success of the messaging and the success of the overall intervention. In the short term, we will look for an improvement over time in the survey results to measure success of the program. In the long term, we will look at the yearly reports on sexual harassment. Initially we will expect to see an increase in reported cases, as the community becomes more aware of the resources available and confident in the Office of Women and Equity to resolve these cases. Overtime we would expect that number to decrease as the policies and education successfully deters this behaviour.

UNILAG plans to continue to work on sexual harassment prevention messaging through the Behavioral Change Communication course since the yearly offering provides an opportunity for an annual review of messages. Using the course to review and update the slogans also allows for reaching and educating a new group of students on the subject of sexual harassment, promote student-led interventions, and awareness resources at the university.
